# Mechanism of Terpene Formation in *Pogostemon cablin* cv. Jing Huoxiang Revealed by Metabolome and Transcriptome Analysis

**DOI:** 10.3390/ijms27156836

**Published:** 2026-07-30

**Authors:** Wei Ma, Xiukun Wan, Ge Yao, Fuli Wang, Hui Jiang

**Affiliations:** State Key Laboratory of Chemistry for NBC Hazards Protection, Beijing 102205, China; weima0224@163.com (W.M.); bzyaoge@163.com (G.Y.); fuliwang@163.com (F.W.)

**Keywords:** *Pogostemon cablin* cv. Jing Huoxiang, transcriptome sequencing, sesquiterpene, biosynthesis, terpene synthase (TPS), metabolomics, transcriptomics, biosynthesis regulation

## Abstract

*Pogostemon cablin* (Blanco) Benth. cv. ‘Jing Huoxiang’, is a valuable medicinal plant widely studied for its aboveground tissues, which are rich in bioactive compounds such as patchouli alcohol. However, systematic investigations into the biosynthesis of sesquiterpenes in its underground parts (roots) remain limited, with several critical knowledge gaps: (1) the metabolic basis of root-specific accumulation of polycyclic sesquiterpenes is unclear; (2) key terpene synthase (TPS) gene resources remain underexplored; and (3) the regulatory network of terpenoid biosynthesis is poorly understood. Addressing these questions is essential for the rational design and efficient production of terpene synthases. In this study, we integrated metabolomic and transcriptomic approaches to systematically characterize terpenoid profiles across different tissues of *P. cablin* and elucidate their biosynthetic regulation. Using GC-MS analysis, we identified distinct terpenoid compositions in roots, stems, leaves, flowers, and glandular trichomes. Notably, patchouli alcohol and pogostone accounted for over 60% of the total volatile oil content, while roots specifically accumulated polycyclic sesquiterpenes such as β-caryophyllene and α-humulene. Through transcriptome sequencing and bioinformatic analysis, we comprehensively annotated the TPS gene family, revealing that the *TPS-a* subfamily (34 genes) was the most abundant in *P. cablin*, with several members exhibiting root-predominant expression. Co-expression network analysis further identified candidate genes encoding potential high-efficiency polycyclic sesquiterpene synthases and uncovered a β-caryophyllene/α-humulene-regulated tertiary metabolic pathway. Our findings not only fill a critical gap in understanding sesquiterpene biosynthesis in the underground tissues of *P. cablin* but also provide a foundation for synthetic biology-based optimization of terpenoid production. This research paves the way for the efficient biosynthesis of sesquiterpenes to meet industrial demands in pharmaceuticals, fragrances, and biofuels.

## 1. Introduction

*Pogostemon cablin* (Blanco) Benth. cv. ‘Jing Huoxiang’, a traditional Chinese medicinal plant with significant pharmacological value [1,2]. This species holds significant value in traditional medicine, where it is employed to dispel aromatic turbidity, alleviate emesis, counteract summerheat syndrome, relieve pain and inflammation, regulate menstruation, and mitigate abdominal discomfort [3,4]. Its primary applications include managing symptoms associated with wind-turbidity syndrome, gastrointestinal distress, vomiting, diarrhea, and cold–dampness-induced fever. Contemporary pharmacological studies indicate that *P. cablin* extracts exhibit notable anticancer and anti-inflammatory activities, with emerging evidence suggesting potential antiviral effects against SARS-CoV-2 [3,4,5,6].

The essential oil, recognized as the primary bioactive component, serves as the fundamental material basis for its therapeutic effects. However, the rapidly increasing market demand presents substantial challenges to current cultivation capacity. The essential oil, recognized as the primary bioactive component, serves as the fundamental material basis for its therapeutic effects. However, the rapidly increasing market demand presents substantial challenges to current cultivation capacity [7,8]. Extensive phytochemical investigations have identified 57 distinct sesquiterpenoids in *P. cablin* essential oil, with patchouli alcohol, pogostone, and patchoulene emerging as the most pharmacologically significant constituents. Notably, the relative abundance of patchouli alcohol and patchoulene has been adopted as crucial quality control parameters for essential oil evaluation [9]. While these sesquiterpenoids demonstrate varying degrees of pharmacological potency, they uniformly exhibit characteristic antimicrobial and anti-inflammatory activities. Despite these advances, current research efforts have predominantly focused on the aerial portions, leaving a substantial knowledge gap regarding the secondary metabolites and their biosynthetic pathways in the root system. This research disparity significantly impedes our understanding of the metabolic foundation, key enzymatic components, and regulatory networks, thereby constraining the targeted development and efficient production of these valuable terpenoids.

From a broader perspective, sesquiterpenes represent the most structurally diverse class of terpenoids in plants, fulfilling critical ecological functions in plant-environment interactions (including defense mechanisms and signaling processes) while possessing substantial commercial value in pharmaceutical, fragrance, and biofuel applications. The biosynthesis of these compounds is governed by sesquiterpene synthases (TPS), a family of enzymes whose genetic diversity and functional specialization directly determine the structural complexity of plant secondary metabolites. Terpene synthases (TPS), ubiquitous in medicinal plants such as *Dendrobium* [10], *Cinnamomum verum* [11], *Mentha piperita* [12], and *Curcuma amaranth* [13], play pivotal roles in terpene cyclization. Although patchoulol synthase has been characterized in *P. cablin*, the functional diversity of other TPS isoforms remains largely unexplored [14].

At the biochemical level, plant terpenoid biosynthesis proceeds through two spatially segregated pathways: the cytosolic mevalonate (MVA) pathway and the plastidial methylerythritol phosphate (MEP) pathway [15]. These parallel routes exhibit distinct product specificities, with the MVA pathway predominantly generating sesquiterpenes and triterpenes, while the MEP pathway primarily supplies precursors for monoterpenes, diterpenes, and tetraterpenes [2]. The biosynthetic cascade initiates with the condensation of dimethylallyl diphosphate (DMAPP) and isopentenyl diphosphate (IPP), catalyzed by specialized prenyltransferases to yield various polyisoprenyl diphosphate intermediates (C10–C25), including geranyl diphosphate (GPP), farnesyl diphosphate (FPP), geranylgeranyl diphosphate (GGPP), and geranylfarnesyl diphosphate (GFPP) [16,17]. These activated precursors serve as substrates for downstream terpene synthases, ultimately giving rise to the structural diversity observed in plant terpenoids.

The advent of multi-omics technologies has revolutionized plant secondary metabolism research. Integrated metabolomic approaches enable comprehensive profiling of metabolic networks across different tissues, while advanced transcriptomic analyses coupled with co-expression network modeling provide powerful tools for identifying key biosynthetic enzymes and predicting their functional relationships. The strategic application of these cutting-edge methodologies holds tremendous promise for elucidating the metabolic signatures and regulatory architecture underlying root-specific sesquiterpene biosynthesis in *P. cablin*, thereby identifying potential targets for synthetic biology applications.

In the present study, we employed an integrated analytical framework combining GC-MS-based metabolomics with full-length transcriptome sequencing to systematically characterize terpenoid metabolic profiles across various *P. cablin* tissues (roots, stems, leaves, flowers, and trichomes). Our comprehensive approach has enabled the identification of TPS gene family members and the selection of candidate genes associated with root-specific sesquiterpene biosynthesis. These findings not only address critical knowledge gaps regarding underground sesquiterpene production in *P. cablin* but also establish a robust theoretical foundation for optimizing terpenoid biosynthesis through synthetic biology approaches to meet growing pharmaceutical and industrial demands. Looking forward, the strategic integration of gene editing and metabolic engineering technologies promises to enable efficient heterologous production of *P. cablin* sesquiterpenes, thereby facilitating their large-scale industrial applications.

## 2. Result

### 2.1. Metabolomic Analysis of Volatile and Terpenoid Metabolites in P. cablin

**Figure 1:**
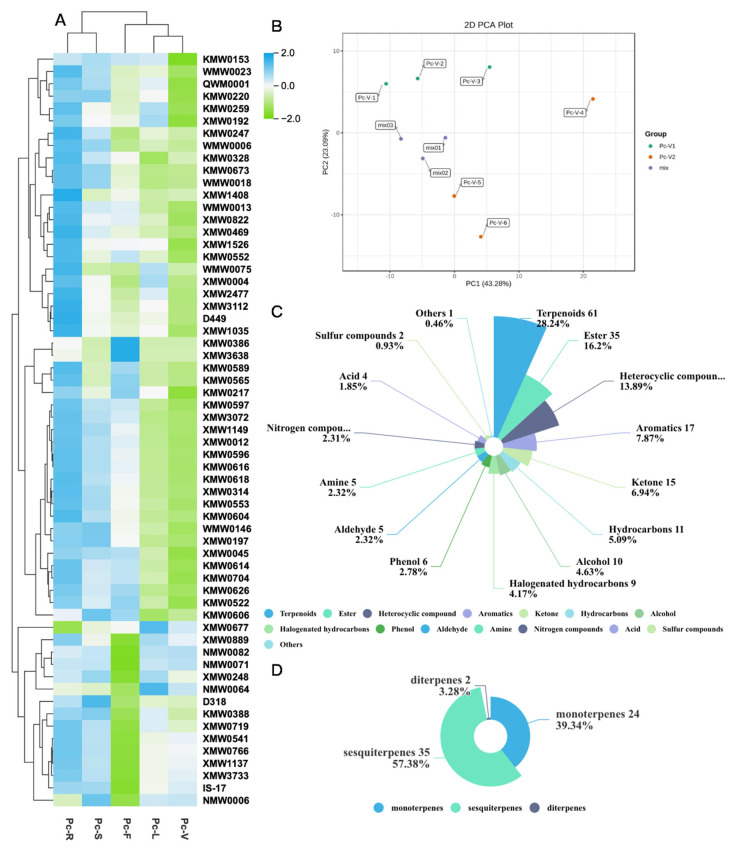
Metabolomic profiling of volatile and terpenoid compounds in *P. cablin*,The figure shows (**A**) GC-MS peaks of essential oils, Pc_R (roots), Pc_S (stems), Pc_L (leaves), Pc_F (flowers), and Pc_W (villosity). (**B**) Principal component analysis (PCA) score plot showing distinct clustering patterns of metabolic profiles from aerial parts (green), underground parts (orange), and mixed samples (purple). (**C**) Relative abundance of different chemical classes in *P. cablin* volatile metabolites. (**D**) Compositional distribution of terpenoid subclasses in *P. cablin*.

Volatile and terpenoid metabolites in *P. cablin* were profiled using metabolomic analysis. Metabolite identification and quantification were performed via mass spectrometry against the MWGC database. MassHunter (Version B.08.00, Agilent Technologies) [18] quantitative software processed raw GC-MS data for chromatographic peak integration and calibration. The GC-MS platform coupled with our custom database detected 217 metabolites, revealing tissue-specific metabolic patterns in patchouli (Figure 1B). Principal Component Analysis (PCA) showed the first two principal components (PC1 = 43.28%, PC2 = 23.09%) cumulatively explained 66.37% of variance, significantly differentiating aerial (Pc-V1) and root tissues (Pc-V2) (*p* < 0.001). Aerial samples (green) distributed across quadrants I and II (PC1: −12 to 6, PC2 > 0), with Pc-V-1 (−12,6) and Pc-V-2 (−4,8) in quadrant II, and Pc-V-3 (6,8) in quadrant I. Dispersion along PC1 axis reflected metabolic differentiation among stems, leaves, and flowers. Root samples (orange) clustered in quadrants I and IV (PC1 > 0), exhibiting high heterogeneity—notably Pc-V-4 (22,5) showed extreme PC1 deviation, while Pc-V-5 (1,−7) and Pc-V-6 (5,−12) occupied quadrant IV, indicating dynamic root metabolism. Quality control mixtures (MIX, purple) tightly aggregated in the negative PC1 region (mix01: −2,−1; mix02: −4,−4; mix03: −9,0), demonstrating metabolic homogenization. This spatial distribution originated from tissue-specific regulatory mechanisms: light-dependent metabolism (e.g., monoterpene synthesis) in aerial parts versus microbe-modulated sesquiterpenoid enrichment in roots. Homogenization in mixtures likely resulted from metabolic complementarity between tissues and oxidation/polymerization reactions.

Detected metabolites encompassed terpenoids, esters, heterocyclic compounds, aromatics, ketones, halogenated hydrocarbons, phenols, amines, nitrogen-containing compounds, aldehydes, organic acids, and sulfur-containing compounds (Figure 1C). Among 61 identified terpenoids, 35 were sesquiterpenes and derivatives (Table 1, Figure 1A), accounting for 57.4% of total terpenoids (Figure 1D). This highlights *P. cablin*’s distinctive sesquiterpenoid biosynthetic capacity, featuring functional C_15_H_24_ sesquiterpenes including α-copaene, β-bourbonene, (-)-γ-elemene, β-caryophyllene, α-humulene, aglaiene, and patchoulol, alongside 24 monoterpenes and 2 diterpenes.

### 2.2. Volatile Metabolite Profiling Reveals Tissue-Specific Biosynthesis in P. cablin Differential Metabolite Analysis

**Figure 2:**
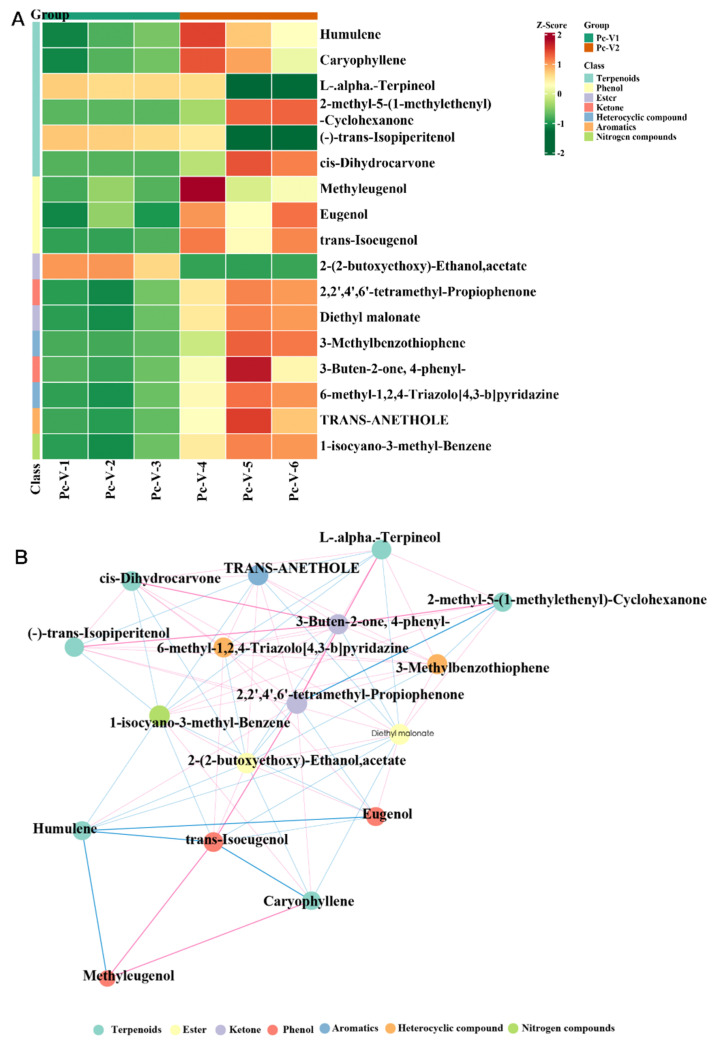
Cluster and correlation analysis of differential volatile metabolites (**A**) Clustering heatmap of differential volatile metabolites; (**B**) Correlation network of differential volatile metabolites. Pc_V1–Pc_V3 represents the aboveground parts, including stems, leaves, flowers, and villosity; Pc_V3–Pc_V6 represents the underground parts, consisting solely of roots.

Based on screening criteria of fold change > 2 and *p* < 0.05, 17 tissue-specific differentially abundant metabolites (DAMs) underwent Unit Variance Scaling (UV) normalization, with distribution patterns visualized through hierarchical clustering (Figure 2A). Results revealed 14 metabolites (82.4%) significantly upregulated in aerial tissues versus only 3 (17.6%) in roots, indicating dominant volatile biosynthesis in aerial organs consistent with their atmospheric exposure requiring enhanced ecological defense. Distinct metabolic compartmentalization was observed: Roots enriched six terpenoids including sesquiterpenes (β-Caryophyllene, α-Humulene) and monoterpenoids/derivatives (L-α-Terpineol, 2-Methyl-5-(1-methylethenyl)-cyclohexanone, (-)-trans-Isopiperitenol, cis-Dihydrocarvone). Aerial tissues accumulated 11 metabolites spanning phenols (Methyl eugenol/Eugenol/trans-Isoeugenol), esters (2-(2-Butoxyethoxy)ethyl acetate/Diethyl malonate), ketones (2,2′,4′,6′-Tetramethylacetophenone [TMAP]/4-Phenyl-3-buten-2-one [BPB]), heterocyclics (3-Methylbenzothiophene), aromatics (trans-Anethole), and nitrogen-containing compounds (6-Methyl-[1,2,4]triazolo[4,3-b]pyridazine/1-Isocyano-3-methylbenzene). This partitioning designates roots as a core terpenoid biosynthesis site where sesquiterpenes mediate root-microbe interactions while monoterpenoids participate in environmental signaling.

Further Pearson correlation analysis constructed a metabolite interaction network (Figure 2B), identifying β-Caryophyllene and α-Humulene as central hubs exhibiting significant correlations with phenols/ketones (|r| > 0.7, *p* < 0.01). Both sesquiterpenes showed negative correlations with Methyl eugenol, trans-Isoeugenol, and 2-(2-Butoxyethoxy)ethyl acetate, but positive correlations with TMAP. Notably, β-Caryophyllene positively correlated with Methyl eugenol (r = 0.73) whereas α-Humulene showed negative correlation (r = −0.68), revealing terpene-phenol metabolic antagonism potentially driven by acetyl-CoA precursor competition or enzymatic regulation (α-Humulene suppressing COMT activity; β-Caryophyllene activating PAL). A three-tiered regulatory cascade was identified: β-Caryophyllene/α-Humulene → TMAP (r = 0.82/0.79) → BPB (r = 0.75) → L-α-Terpineol (r = 0.71), potentially enabling dynamic metabolic flux redistribution. Extensive cross-class interactions (e.g., terpenoid-phenol/ketone correlations) and strong intra-phenol positive correlations collectively demonstrate high integration within secondary metabolic networks.

### 2.3. RNA Sequencing and Transcriptome Assembly

Transcriptome sequencing analysis was conducted on 15 experimental samples representing distinct tissues of *P. cablin* during full-bloom stage, including roots (Pc-R), stems (Pc-S), leaves (Pc-L), flowers (Pc-F), and glandular trichomes (Pc-W). Following raw data filtering, sequencing error rate verification, and GC content distribution assessment, high-quality clean reads were obtained for subsequent analyses. A total of 103.77 Gb Clean Data was generated, with each sample yielding ≥6 Gb of Clean Data. The Q30 base percentage reached ≥91% across all samples. As detailed in Table 2, comprehensive quality assessment demonstrated: the overall Clean Data volume reached 103.77 Gb, all samples contained ≥6 Gb Clean Data, Q30 base percentages were ≥91%, Q20 base percentages exceeded 96%, sequencing error rates were controlled within 0.02% to 0.03%, and GC content distributions ranged from 47% to 51%. These metrics confirm compliance with standard quality requirements for plant transcriptome sequencing. The high-quality dataset establishes a robust foundation for subsequent gene functional annotation, differential expression analysis, and metabolic pathway investigation.

### 2.4. Identification of DEGs in Different Tissues

**Figure 3:**
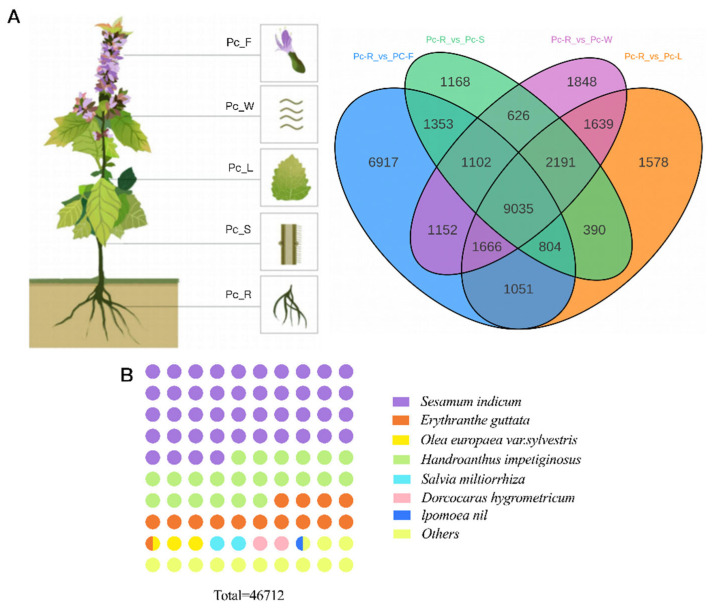
Analysis of differential genes in different tissues (**A**) Different tissues of patchouli (**B**) Differences in transcriptomes of different tissues.

Cross-tissue expression profiling analysis in *P. cablin* identified significantly differentially expressed genes (DEGs): Comparative analysis revealed 1168 DEGs between roots (Pc_R) and stems (Pc_S), 1578 DEGs between roots and leaves (Pc_L), 6917 DEGs between roots and flowers (Pc_F) (significantly higher than other comparisons), and 1848 DEGs between roots and glandular trichomes (Pc_W) (Figure 3A). Homology analysis of *P. cablin* transcripts against the NCBI non-redundant protein (NR) database revealed the following cross-species similarities (Figure 3B): *Sesamum indicum* (sesame) showed the highest sequence similarity at 45.33%, indicating closest phylogenetic relationship. As fellow Lamiales species, this suggests retention of shared ancestral genetic features. Functional parallels were observed in lipid metabolism genes, secondary metabolite biosynthesis pathways, and drought-responsive proteins, reflecting convergent evolution under specific environmental pressures and implying analogous adaptive traits. *Handroanthus impetiginosus* ranked second with 22.06% similarity, demonstrating substantial transcriptional commonalities. Potential conservation exists in lignin biosynthesis pathways, defense-related proteins, and anthocyanin metabolism genes. Ecological adaptations suggest both species may have developed similar stress-resistance mechanisms through shared secondary metabolic regulatory networks. *Erythranthe guttata* accounted for 14.03% similarity, indicating conserved genetic information and physiological functions. Other relevant species (collectively 12.11%) included *Olea europaea* (wild olive), *Salvia miltiorrhiza* (danshen), *Dorcoceras hygrometricum*, and *Ipomoea nil* (morning glory). Similarity to *S. miltiorrhiza* suggests potential shared terpenoid biosynthesis pathways and analogous medicinal compounds. Parallels with wild olive imply convergent evolution of drought-resistance mechanisms in secondary metabolism. These comparative results provide diverse biological insights for further investigation of *P. cablin*’s biological functions and evolutionary relationships.

### 2.5. Phylogenetic and Functional Characterization of PcTPS

A phylogenetic tree constructed from amino acid sequences provides robust evidence for the classification of terpene synthase (TPS) proteins into five distinct subfamilies: *TPS-a*, *TPS-b*, *TPS-c*, *TPS-e/f*, and *TPS-g*. Within these proteins, certain conserved domains have been identified, notably the DDXXD motif, which is highlighted in yellow, and the NSE/DTE motif, highlighted in orange. The *TPS-a* family, specifically, is known to encode sesquiterpene synthases and is distinguished by several conserved structural features, including the RR(X)8W motif in addition to the DDXXD and NSE/DTE motifs. However, it is crucial to acknowledge that the RR(X)8W motif, although commonly linked to sesquiterpene synthases, is not completely preserved across all TPS proteins, indicating some variability. Within the species *P. cablin*, the *TPS-a* subfamily emerges as the most abundant, containing a notable 34 genes. This is followed by the *TPS-b* subfamily, which consists of 17 genes, and the *TPS-c* subfamily, which has a comparatively smaller representation with only 9 genes. Interestingly, this study did not identify any genes from the *TPS-e/f* and *TPS-g* subfamilies, pointing to a potential gap in their presence within this organism. Additionally, a differential expression analysis conducted across various tissue types has uncovered significant disparities in both the localization and expression levels of these TPS genes. These findings suggest that the roles of TPS genes may vary widely across different tissues within *P. cablin*, indicating their possible involvement in diverse biological functions and metabolic processes tailored to specific tissue environments.

**Figure 4:**
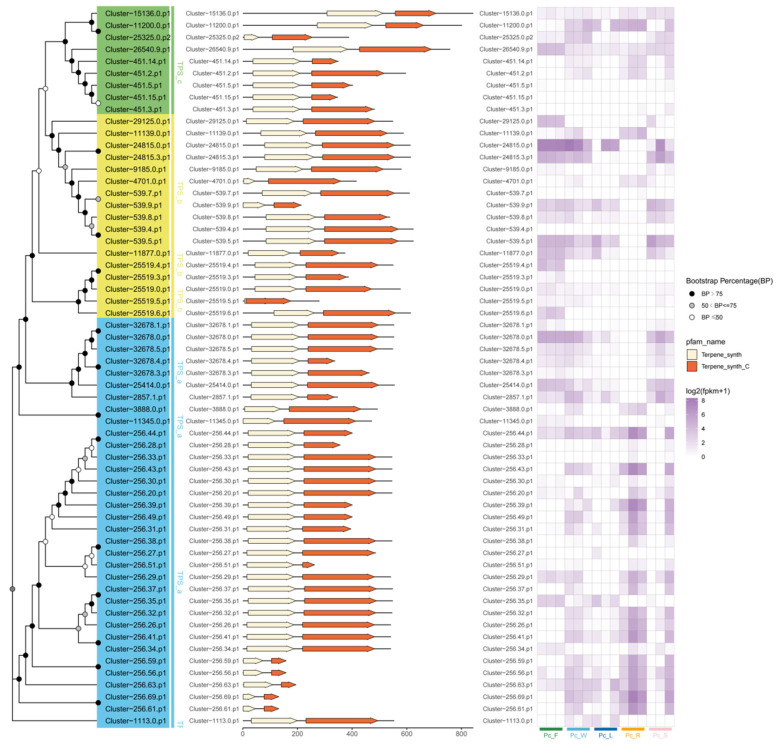
Phylogenetic tree results of the *PcTPS* gene family and representative terpene synthases.

Transcriptomic analysis identified 60 terpene synthase (TPS) genes in *P. cablin* (Figure 4), encoding enzymes harboring the conserved DDXXD (divalent metal-binding) and NSE/DTE (substrate-binding/catalytic) motifs. Phylogenetic reconstruction resolved these TPS genes into three principal subfamilies: The *TPS-a* subfamily (34 genes; blue), predominantly encoding sesquiterpene synthases, constituted the largest group and subdivided into three distinct clades (I-III), suggesting functional diversification. Clade I, dominated by Cluster-256 members (e.g., Cluster-256.44.p1, Cluster-256.28.p1), exhibited high sequence conservation around the catalytic motifs and adjacent loop regions, implying functional redundancy potentially arising from recent gene duplications. Clade II, primarily comprising Cluster-32678 members (e.g., Cluster-32678.1.p1, Cluster-32678.0.p1), retained conserved catalytic cores but displayed significant divergence in flanking flexible loop regions (“looTPS” domains) that modulate substrate binding and product specificity, indicating potential functional divergence. Clade III contained phylogenetically dispersed members (e.g., Cluster-1113.0.p1, Cluster-2857.1.p1, Cluster-11345.0.p1) with conserved catalytic domains but variable N-/C-termini, suggesting specialization in specific sesquiterpenoid pathways. The *TPS-b* subfamily (17 genes; yellow), primarily monoterpene synthases, retained the DDXXD motif but lacked the characteristic RR(X)_8_W motif, potentially representing an ancient evolutionary lineage. The *TPS-c* subfamily (9 genes; green), encoding geranylgeranyl diphosphate synthases (GGPPS) for diterpene precursor biosynthesis, represented the smallest subfamily, consistent with patterns observed across plant taxa. Conserved domain analysis confirmed universal presence of essential “Terpene_synth” and “Terpene_synth_C” Pfam domains (Figure 4 middle panel). Tissue-specific expression profiling (log_2_[TPM + 1]; Figure 4, right panel) revealed pronounced spatial regulation: *TPS-a* genes showed dominant expression in roots (Pc_R), aligning with sesquiterpene accumulation and validating roots as the primary biosynthetic site; *TPS-b* genes exhibited peak activity in flowers (Pc_F) with minimal root expression, indicating floral specialization for monoterpene production; *TPS-c* genes displayed constitutive expression across all tissues, suggesting ubiquitous diterpene precursor synthesis. This integrated analysis highlights the TPS family’s complexity in *P. cablin*, with *TPS-a* dominance and root-specific sesquiterpene synthase expression reflecting the sesquiterpene-rich medicinal properties of patchouli roots, while floral-enriched *TPS-b* expression correlates with aroma biosynthesis. These findings provide a foundation for targeted functional characterization of key synthases (particularly within *TPS-a* clades) and facilitate molecular breeding for enhanced terpenoid production.

### 2.6. DEGs Involved in Sesquiterpene Biosynthesis

**Figure 5:**
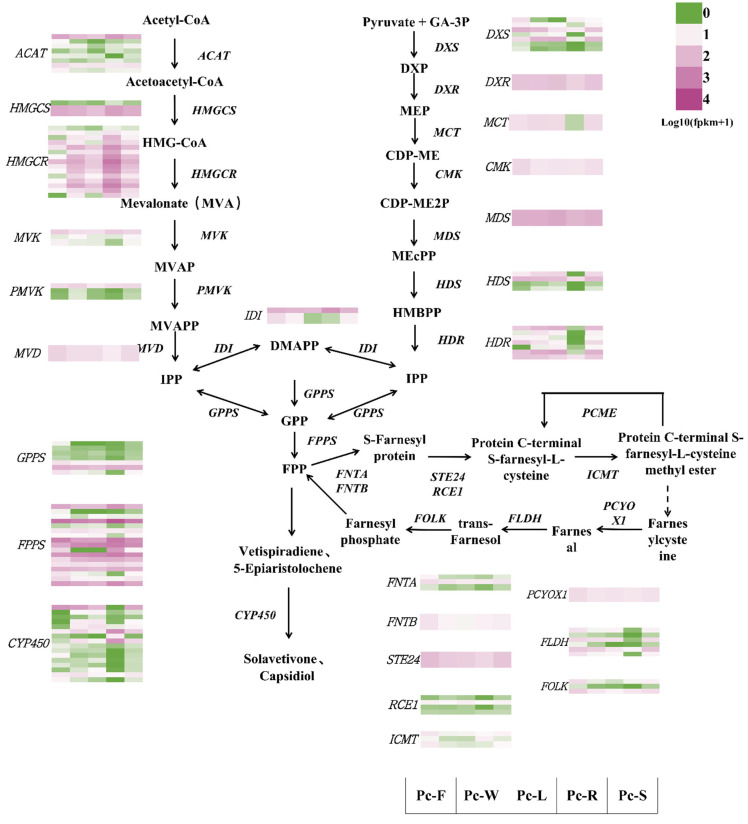

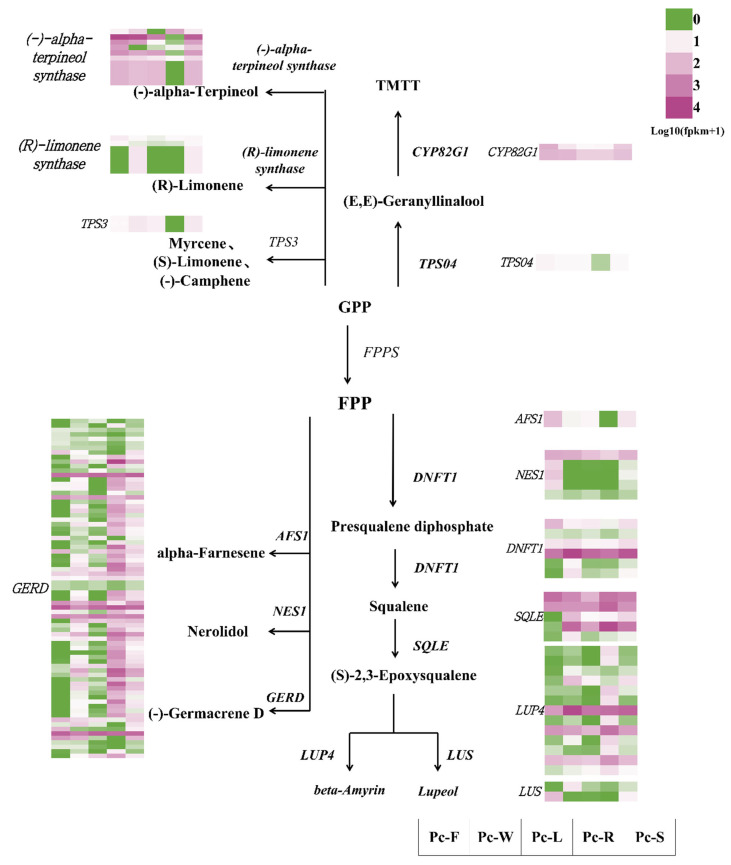
The biosynthetic pathways that lead to the formation of sesquiterpenoids are characterized by a sophisticated interplay of various enzymes. One of the pivotal enzymes in this biosynthetic process is acetyl-CoA-acetyltransferase (AACT), which serves as the entry point by enabling the transfer of acetyl groups to initiate the pathway. Following this initial step, hydroxymethylglutaryl-CoA synthase (HMGS) and 3-hydroxy-3-methylglutaryl-CoA reductase (HMGR) are essential components of the mevalonate pathway. These enzymes are instrumental in generating intermediate compounds that are fundamental for the successful production of sesquiterpenoids. Additionally, the sequential phosphorylation of mevalonate precursors is facilitated by enzymes such as mevalonate kinase (MVK) and phosphomevalonate kinase (PMK). These enzymes further modify the precursors, transforming them into their active forms, which are critical for subsequent biochemical reactions in the pathway. In parallel, the non-mevalonate pathway is exemplified by key enzymes including 1-deoxy-D-xylulose-5-phosphate synthase (DXS) and 1-deoxy-D-xylulose-5-phosphate reductoisomerase (DXR). These enzymes play a crucial role in the synthesis of isopentenyl pyrophosphate (IPP) and dimethylallyl pyrophosphate (DMAPP), both of which serve as vital building blocks for the creation of terpenoids. The biosynthetic journey continues with the actions of 2-C-methyl-D-erythritol 4-phosphate cytidylyltransferase (MCT) and 2-C-methyl-D-erythritol 2,4-cyclic diphosphate synthase (MDS). These enzymes are involved in transforming intermediates into cyclic diphosphate forms, which are further necessary for the synthesis of sesquiterpenoids. As the pathway progresses, subsequent enzymes, including 4-hydroxy-3-methylbut-2-en-1-yl diphosphate synthase (HDS) and isopentenyl diphosphate delta isomerase (IDI), ensure the production of essential precursors that are vital for the elongation process. Finally, the synthesis of diverse sesquiterpenoids is accomplished through the combined actions of geranyl diphosphate synthase (GPPS), farnesyl diphosphate synthase (FPPS), and geranylgeranyl pyrophosphate synthase (GGPPS). This intricate process culminates with the activity of terpenoid synthase (TPS), which acts to convert these precursors into an extensive array of bioactive sesquiterpenoid compounds, showcasing the remarkable complexity and efficiency of the biosynthetic pathways involved.

KEGG pathway analysis revealed that terpenoid biosynthesis in *P. cablin* primarily occurs via the Mevalonate (MVA) and Methylerythritol phosphate (MEP) pathways. Key MVA pathway genes (ACAT, HMGCS, HMGCR, MVK, PMVK, MVD, GPTPS, FPTPS, CYP450, GERD), crucial for sesquiterpene and triterpene synthesis, were significantly expressed (Figure 5). Notably, HMGCS and GERD (Germacrene D synthase) exhibited significantly higher expression in roots compared to other tissues, indicating roots are likely the primary site for sesquiterpene biosynthesis. Conversely, CYP450 and GERD were downregulated in flowers and leaves, potentially reflecting a metabolic shift in these tissues towards monoterpene or volatile terpene synthesis. Key MEP pathway genes (DXS, DXR, MCT, CMK, MDS, HDS, HDR), primarily involved in monoterpene synthesis, were expressed at lower levels than MVA genes but still significantly, suggesting monoterpene synthesis in patchouli may partially rely on the MEP pathway, though its overall contribution is smaller. The lower expression of MEP pathway genes may correlate with the plant’s relatively low monoterpene yield, as its terpenoid metabolism appears more biased towards MVA-driven sesquiterpene and triterpene biosynthesis.

Accumulation of sesquiterpenes occurs predominantly in roots, consistent with the high expression of MVA pathway genes (especially HMGCS and GERD) there. Thirteen monoterpene synthase genes were identified: seven (-)-α-Terpineol synthases were all upregulated in flowers and stems, while four were downregulated in roots, suggesting flowers and stems are major sites for α-Terpineol synthesis. Four (R)-Limonene synthases were all upregulated in flowers but downregulated in leaves, indicating Limonene is primarily synthesized in flowers and less metabolized in leaves. Three TPS3 genes are predicted to synthesize Myrcene, (S)-Limonene, and (-)-Camphene, requiring further in vivo validation.

A total of 84 sesquiterpene synthase genes were identified. Seventy-four GERD genes, catalyzing the synthesis of (-)-Germacrene D from FPP, were mostly upregulated in roots and stems but downregulated in flowers and leaves, suggesting Germacrene D primarily accumulates in roots. Five AFS1 (α-Farnesene synthase) genes (catalyzing α-Farnesene synthesis) require further validation of their expression patterns. Five NESI (Nerolidol synthase) genes, catalyzing Nerolidol synthesis, showed greater upregulation in flowers, potentially contributing to floral volatile synthesis. Germacrene D’s high expression in roots aligns with metabolomic data showing root enrichment of its derivative β-Caryophyllene. Nerolidol’s high floral expression suggests a role in floral scent or defense.

Six triterpene synthase genes were identified, including five DNFT1 (Difarnesyltransferase), thirteen SQLE (Squalene epoxidase), and thirteen LUP4 (β-Amyrin synthase) genes. DNFT1 catalyzes the synthesis of squalene precursors; one gene was upregulated in all tissues, while others were downregulated in glandular trichomes, leaves, and roots, indicating tissue-specific squalene synthesis. SQLE genes were all upregulated in glandular trichomes and significantly upregulated in leaves, roots, and stems, suggesting active triterpene synthesis in these tissues. LUP4 was most upregulated in roots but downregulated in flowers and leaves, indicating β-Amyrin likely accumulates mainly in roots. Three LUS (Lupeol synthase) genes were all downregulated in leaves and roots but upregulated in flowers and glandular trichomes, suggesting tissue-specific lupeol synthesis. Triterpenes like β-Amyrin and lupeol may function in plant defense or membrane stability, with their high expression in roots and glandular trichomes possibly related to protective roles. Upregulation of LUS in flowers may influence floral volatile synthesis.

Six other genes were identified: Three CYP82G1 genes, catalyzing the synthesis of TMTT (4,8,12-trimethyltrideca-1,3,7,11-tetraene) from (E,E)-Geranyllinalool, were upregulated in all tissues, suggesting TMTT may act as a broad-spectrum defense compound. Three TPS04 genes, catalyzing (E,E)-Geranyllinalool synthesis from GPP, were downregulated in roots but upregulated in other tissues, possibly related to the root’s bias towards sesquiterpene synthesis.

In summary, sesquiterpene synthesis in *P. cablin* primarily relies on the MVA pathway, with roots serving as the main site for sesquiterpenes like Germacrene D and β-Caryophyllene. Functional validation of key TPS genes (GERD, AFS1, NESI) is needed to confirm their catalytic products. High expression of monoterpene synthase genes in flowers and stems may relate to the ecological functions (pollination, defense) of volatile secondary metabolites. Triterpene synthase genes (SQLE, LUP4) are highly expressed in glandular trichomes and roots, potentially involved in defense or membrane stability. The ubiquitous upregulation of CYP82G1, synthesizing TMTT, indicates TMTT may be a significant defensive metabolite in *P. cablin*.

### 2.7. Validation of Expression of Tissue-Specific Candidate Genes Related to Sesquiterpene Biosynthesis by qRT-PCR

**Figure 6:**
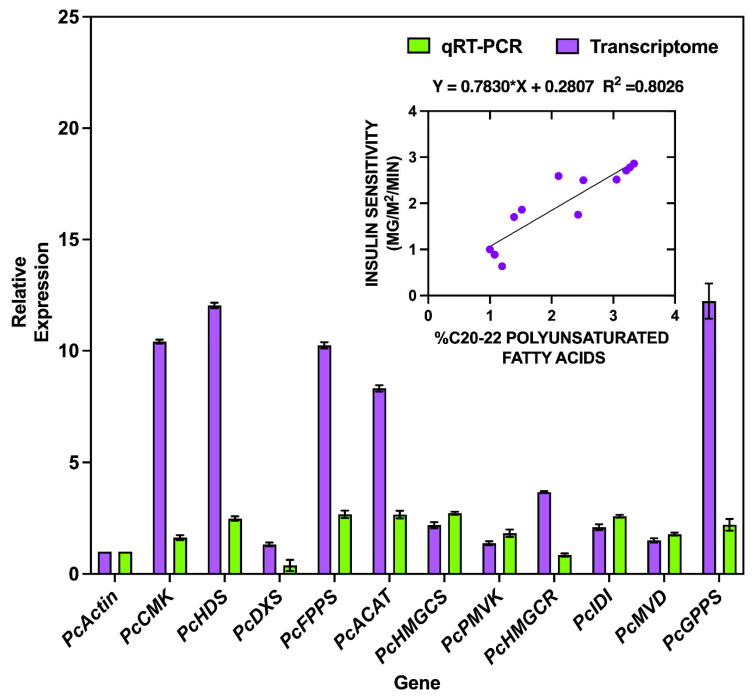
qRT-PCR validates the expression of genes related to sesquiterpene biosynthesis.

To validate the reliability of transcriptome sequencing data, this study randomly selected 10 key terpenoid biosynthesis-related genes for qRT-PCR verification (Figure 6). The genes included: PcAACT (acetyl-CoA C-acetyltransferase), PcHMGS (3-hydroxy-3-methylglutaryl-CoA synthase), PcHMGR (3-hydroxy-3-methylglutaryl-CoA reductase), PcPMK (phosphomevalonate kinase), PcFPTPS (farnesyl diphosphate synthase), PcDXS (1-deoxy-D-xylulose-5-phosphate synthase), PcCMK (4-(cytidine 5′-diphospho)-2-C-methyl-D-erythritol kinase), and PcHDS (4-hydroxy-3-methylbut-2-enyl diphosphate synthase), with PcActin serving as the reference gene.

Validation analysis revealed that qRT-PCR expression trends substantially aligned with transcriptome sequencing data, demonstrating a coefficient of determination (R^2^) of 0.8026. This strong correlation confirms the biological accuracy of the RNA-seq data and establishes a reliable foundation for subsequent terpenoid biosynthesis gene mining and functional analysis. Examination of gene expression patterns showed consistently low expression of PcActin by both methods, while genes such as PcHDS, PcFPPS, and PcGPPS exhibited high expression in transcriptome data with corresponding moderate-to-high expression levels in qRT-PCR. Although transcriptome data generally displayed higher relative expression values than qRT-PCR, both methods consistently captured the relative expression patterns across genes.

Biologically, the qRT-PCR validation not only confirms transcriptome data reliability but more importantly provides a robust basis for functional gene discovery and metabolic pathway elucidation. Notably, the concordant validation of highly expressed genes in transcriptome data (e.g., PcHDS, PcFPPS, PcGPPS) through qRT-PCR provides compelling evidence for the biological reliability and accuracy of this study’s transcriptome dataset.

## 3. Discussion

This study presents the first integrated metabolomic and transcriptomic analysis systematically revealing the tissue-specific regulatory mechanisms of terpenoid biosynthesis in *P. cablin*. Metabolic profiling demonstrated that underground organs (roots) specifically accumulate polycyclic sesquiterpenes (e.g., β-caryophyllene, α-humulene) at levels up to 2.5-fold higher than in aerial organs, whereas aerial parts predominantly contain monoterpenes and phenolic compounds. This distribution pattern holds significant ecological implications: β-caryophyllene, a known defensive compound against aboveground herbivores [19], accumulates substantially in roots, suggesting its potential role in resisting soil pathogens or nematodes. This reflects an evolutionary adaptation strategy to underground biotic stress. A similar mechanism was observed in *Atractylodes lancea*, where sesquiterpene synthase gene expression in rhizomes exhibits chemotype specificity. Key genes regulating β-eudesmol and hinesol synthesis show significantly higher expression in the Dabieshan chemotype compared to the Maoshan chemotype, directly driving differential sesquiterpene enrichment [20].

Transcriptome analysis further elucidated the molecular regulatory basis: Phylogenetic analysis revealed that the *TPS-a* subfamily (containing 34 members) dominates terpenoid biosynthesis in patchouli. Clade I genes (e.g., Cluster-256.44.p1) exhibit root-specific high expression (*p* < 0.001), strongly correlating with sesquiterpene accumulation patterns. While these genes harbor conserved DDXXD and RR(X)_8_W domains, Clade II members display significant variation in the “looTPS” flexible loop region, potentially modulating substrate binding specificity through conformational changes. Metabolic pathway analysis confirmed the cytosolic mevalonate (MVA) pathway as the core engine for sesquiterpene synthesis: Key genes (GERD, HMGCS) were significantly upregulated in roots, and MVA pathway gene expression levels (e.g., HMGCR) markedly exceeded those of the methylerythritol phosphate (MEP) pathway (e.g., DXS). This finding has significant applied value—the MVA pathway is widely utilized in microbial cell factories to replace plant extraction. For instance, reconstructing carbon flux and expressing β-caryophyllene synthase in Saccharomyces cerevisiae [21], enables efficient heterologous sesquiterpene production [20].

Notably, functional predictions of sesquiterpene synthases often deviate from actual product expression. In natural plant systems, the genetic expression of β-caryophyllene synthase displays marked imbalance: The compound was detected in only 11 out of 61 germplasms [19], indicating its synthesis is regulated by specific genetic loci and exhibits high germplasm specificity. This disconnect arises from the combined effects of genetic background differences, host physiological states, and limitations in enzymatic models, underscoring that function prediction based on homologous genes requires experimental validation (e.g., enzymatic activity assays in plant or microbial chassis systems). This study has limitations: Although co-expression networks predicted tertiary metabolic pathways for β-caryophyllene/α-humulene (e.g., β-Caryophyllene → TMAP → BPB → L-α-Terpineol), the enzyme cascade reactions require in vitro validation. Furthermore, the epigenetic regulatory mechanisms governing sesquiterpene synthesis in underground organs remain unexplored. Future work will focus on optimizing MVA-MEP hybrid pathways using dynamic regulatory systems and reconstructing patchouli sesquiterpene synthesis networks in microbial chassis to establish sustainable production platforms for pharmaceutical, fragrance, and bioenergy applications.

Lamiaceae plants commonly exhibit significant expansion of the TPS gene family, which is closely associated with the diversity of their secondary metabolites [22]. In *P. cablin*, the *TPS-a* subfamily (containing 34 members) demonstrates relatively conserved functionality and dominates terpenoid biosynthesis. While most studies rely on genomic predictions and in vitro enzymatic assays, in vivo functional validation (e.g., gene knockout) remains lacking. The divergence in enzymatic activity directly determines chemotypic differences relevant to medicinal/fragrance applications. Future research should integrate structural biology and synthetic biology approaches to unlock its application potential.

## 4. Methods and Materials

### 4.1. Plant Material

Seeds of *P. cablin* were sourced from Zhanjiang, Guangdong Province, China, and cultivated under standardized conditions in controlled growth chambers at a Beijing laboratory. Plants were maintained at 25 °C with LED-regulated long-day photoperiods (16 h light/8 h dark). At peak flowering stage (approximately 8-month-old plants), systematic tissue sampling was conducted: For RNA-Seq analysis, leaves, stems, flowers, roots, and villosity (epidermal glandular trichomes) were collected with three biological replicates per tissue type, sampled from distinct regions of individual plants; for qRT-PCR validation, identical tissue types and replication strategies were employed; for volatile compound analysis, fresh tissues were harvested in triplicate as independent biological replicates. All samples were immediately flash-frozen in liquid nitrogen. Primary aliquots were processed for analysis while backup aliquots were preserved at −80 °C in ultra-low temperature freezers.

### 4.2. Metabolite Sample Extraction and Analysis

Tissue samples from five types were pulverized in liquid nitrogen and homogenized by vortexing. Aliquots (1 g solid or 1 mL liquid homogenate) were transferred to 20 mL headspace vials (Agilent, Palo Alto, CA, USA) containing saturated NaCl solution to inhibit enzymatic activity, supplemented with 10 μL of internal standard solution (50 μg/mL), and sealed with crimp-top caps fitted with TFE-silicone septa (Agilent). Automated headspace solid-phase microextraction (HS-SPME) was performed using a 120 µm DVB/CAR/PDMS fiber (Supelco, Bellefonte, PA, USA/Agilent Technologies, Santa Clara, CA, USA). Samples were equilibrated at 100 °C with agitation for 5 min prior to 15 min headspace extraction at 100 °C. New fibers were conditioned for 2 h at 250 °C before initial use and reconditioned for 5 min at 250 °C before each sampling. Thermal desorption occurred in the GC injector at 250 °C for 5 min under splitless mode with a 3.5 min solvent delay.

GC-MS analysis was conducted on an Agilent 8890 GC coupled to a 5977B MS (Agilent Technologies). Separation utilized a DB-5MS capillary column (30 m × 0.25 mm × 0.25 μm; Agilent J&W Scientific) with high-purity helium (≥99.999%) as carrier gas at a constant flow rate of 1.2 mL/min. The oven temperature program initiated at 40 °C (hold 3.5 min), ramped at 10 °C/min to 100 °C, then 7 °C/min to 180 °C, and finally 25 °C/min to 280 °C (hold 5 min). The transfer line, ion source, and quadrupole temperatures were maintained at 280 °C, 230 °C, and 150 °C, respectively. Electron impact ionization (70 eV) and full-scan acquisition (*m*/*z* 50–500 amu, 1 scan/s) were employed.

Metabolite identification combined spectral matching against the MWGC/NIST databases with retention index verification. Quantitation was performed in MassHunter Version B.10.00 software [18] using characteristic quantifier ions for peak integration and calibration. Multivariate analysis in R included: (1) unsupervised PCA (prcomp) on unit variance-scaled data to assess metabolic variation; (2) HCA with dendrograms (ComplexHeatmap) and sample-wise Pearson correlation heatmaps (cor); (3) OPLS-DA (MetaboAnalystR) for identifying significantly differentially abundant metabolites (VIP ≥ 1 and |log2FC| ≥ 1) after log2 transformation and mean-centering, validated by 200 permutation tests; and (4) functional annotation via KEGG Compound database mapping to KEGG Pathways, with Metabolite Set Enrichment Analysis (MSEA) significance determined by hypergeometric test *p*-values.

### 4.3. RNA Extraction and Sequencing Library Construction

RNA integrity and potential DNA contamination were assessed via agarose gel electrophoresis using an improved CTAB method [23,24]. RNA purity was determined by OD260/280 and OD260/230 ratios measured with a NanoPhotometer spectrophotometer (Implen). Precise RNA quantification was performed using a Qubit 2.0 Fluorometer (Thermo Fisher Scientific), and RNA integrity was verified with an Agilent 2100 Bioanalyzer to ensure compliance with library construction standards.

Next-generation sequencing (NGS) was conducted on the DNBSEQ platform: mRNA was enriched and purified using oligo (dT) magnetic beads, followed by fragmentation with proprietary lysis buffer. First-strand cDNA synthesis was initiated by reverse transcriptase with random hexamer primers, then second-strand synthesis was completed. After end repair, poly-A tailing, and adapter ligation with RNA index sequences, cDNA fragments were amplified by PCR and quality-checked using the Agilent 2100 system. Finally, double-stranded PCR products were denatured and circularized via adapter oligonucleotides for library preparation.

Full-length transcriptome sequencing employed the PacBio platform workflow: First- and second-strand cDNA synthesis was performed using the SMARTer PCR cDNA Synthesis Kit (Clontech) with SMARTScribe™ Reverse Transcriptase. Large-scale PCR amplification was conducted with PrimeSTAR GXL DNA Polymerase and 5′-PCR Primer IIA to generate sufficient double-stranded cDNA. Size-fractionated cDNA fragments were purified and enriched for library construction, with sequencing analysis completed on the PacBio Sequel platform at Metware Biotechnology (Wuhan, China).

### 4.4. Gene Annotation and Differential Gene Expression Analysis

For putative gene function prediction, the final full-length isoforms were aligned against multiple databases using BLAST software (v2.2.23) [25], including: NCBI non-redundant protein sequences (NR; ftp://ftp.ncbi.nlm.nih.gov/blast/db/, accessed on 15 May 2023), NCBI non-redundant nucleotide sequences (NT; ftp://ftp.ncbi.nlm.nih.gov/blast/db/, accessed on 6 November 2025), SwissProt (https://www.uniprot.org/uniprotkb?reviewed=true, accessed on 6 November 2025), Kyoto Encyclopedia of Genes and Genomes (KEGG; http://www.genome.jp/kegg, accessed on 6 November 2025), and Eukaryotic Orthologous Groups (KOG; ftp://ftp.ncbi.nih.gov/pub/COG/KOG/, accessed on 6 November 2025). Gene Ontology (GO; http://www.geneontology.org, accessed on 6 November 2025) annotations and functional classifications were obtained through Blast2GO using NR annotation results. All clean reads from samples were mapped to the transcriptome assembly using Bowtie2 [26]. Read counts and gene expression levels were normalized via FPKM (Fragments Per Kilobase per Million mapped reads) with RSEM software (v1.3.3) [27]. Genes exhibiting a Q-value (adjusted *p*-value) < 0.001 and absolute fold change ≥ 2 (as identified by DESeq2) [28] were designated as differentially expressed genes (DEGs).

Raw sequencing data have been deposited in the NCBI Sequence Read Archive (SRA) under BioProject accession PRJNA1344881 (https://www.ncbi.nlm.nih.gov/sra/PRJNA1344881, accessed on 6 November 2025). Metabolomics data are available in the EMBL-EBI MetaboLights repository under accession MTBLS13122.

### 4.5. Sequence Analysis and Identification of TPS Gene Family

Clean reads from all samples were aligned to the transcriptome assembly using Bowtie2. Gene expression quantification and normalization were performed with RSEM software based on the FPKM (Fragments Per Kilobase of transcript per Million mapped reads) method. Differentially expressed genes (DEGs) were screened using the DESeq2 package [28] with selection thresholds set at a corrected *p*-value (Q-value) < 0.001 and absolute fold change ≥ 2 (i.e., |log2FC| ≥ 1).

Identification of *P. cablin* TPS genes was conducted through the following pipeline: Multi-species TPS gene family sequences were retrieved from the NCBI database, followed by homology search via local BLAST in BioEdit software (https://thalljiscience.github.io/, accessed on 6 November 2025) (E-value cutoff = 1.0 × 10^−50^). Multiple sequence alignment of full-length TPS genes was performed using BioEdit. Tertiary structures of PcTPSs proteins were visualized and analyzed using SwissModel (http://swissmodel.expasy.org/, accessed on 6 November 2025) and PyMOL v2.2.0 [29] (https://pymol.org/2/, accessed on 6 November 2025). Phylogenetic tree construction was executed in MEGA 7.0 [30] software employing the Neighbor-Joining method with 1000 bootstrap replicates, and the final phylogenetic tree was generated using the iTOL [31] online tool (https://itol.embl.de/, accessed on 6 November 2025) [32].

### 4.6. Real-Time Quantitative Reverse Transcription PCR

Real-time quantitative reverse transcription PCR (qRT-PCR) was utilized to confirm the reliability of the sequencing results obtained in the studies [33,34,35]. A random selection of 10 key genes involved in the terpenoid biosynthesis pathway of *P. cablin* were validated. Subsequently, the tissue-specific expression patterns of terpene synthase (TPS) genes were analyzed via quantitative reverse transcription PCR (qRT-PCR). Total RNA was extracted from different *P. cablin* tissue samples and reverse-transcribed into first-strand cDNA. Three technical replicates were performed for each sample, with gene expression quantified using the 2^−ΔΔCt^ method [36] and normalized against the actin reference gene. Amplification reactions were conducted using an ABI StepOnePlus™ Real-Time PCR System (Applied Biosystems, Foster City, CA, USA) with Ruiboxingde 2× RePcab Green PCR Fast Mix (Beijing, China).

## 5. Conclusions

This study presents the first integrated metabolomic and transcriptomic analysis to systematically elucidate the tissue-specific regulatory mechanisms of terpenoid biosynthesis in *P. cablin*. Metabolic profiling revealed that underground organs (roots) specifically accumulate polycyclic sesquiterpenes (e.g., β-caryophyllene, α-humulene) at levels up to 2.5-fold higher than aerial organs, whereas aerial parts are dominated by monoterpenes and phenolic compounds, reflecting an evolutionary adaptation strategy to underground biotic stress (e.g., defense against soil pathogens). At the molecular level, the *TPS-a* subfamily (34 members) dominates terpenoid biosynthesis, with Clade I genes (e.g., Cluster-256.44.p1) exhibiting root-specific high expression (*p* < 0.001), strongly correlating with sesquiterpene accumulation. Variations in the “looTPS” flexible loop of Clade II members may modulate substrate specificity through conformational changes. Pathway analysis confirmed the cytosolic mevalonate (MVA) pathway as the core engine for sesquiterpene synthesis, evidenced by significant upregulation of key genes (GERD, HMGCS, HMGCR) in roots and their markedly higher expression compared to the methylerythritol phosphate (MEP) pathway (e.g., DXS). These findings hold substantial applied significance: reconstructing the MVA pathway in microbial chassis (e.g., *Saccharomyces cerevisiae*) enables efficient heterologous sesquiterpene production, offering sustainable strategies for pharmaceutical, fragrance, and bioenergy industries. Limitations remain: functional predictions of sesquiterpene synthases frequently deviate from actual product expression, necessitating enzymatic validation and exploration of epigenetic regulation. Future work will optimize MVA-MEP hybrid pathways via dynamic regulatory systems to establish microbial cell factories for patchouli sesquiterpenes.

## Figures and Tables

**Table 1:** Terpenoids obtained from different tissues of *P. cablin*.

Index	Formula	RI	NIST_RI	Compounds	CAS	Match Factor
IS-17	C_13_H_17_D_3_O	1479.29	1240	[2H3]-beta-Ionone	18252-44-3	98.1
XMW2477	C_20_H_40_O	2108.24	2114	Phytol	87-44-5	98.8
XMW3112	C_20_H_40_O	1942.06	1948	Isophytol	483-76-1	96.0
XMW0822	C_15_H_24_	1484.86	1432	Tricyclo [4.4.0.02,7]decane, 1-methyl-3-methylene-8-(1-methylethyl)-, stereoisomer	24703-35-3	93.9
KMW0565	C_15_H_24_	1425.61	1467	Caryophyllene	514-51-2	98.4
KMW0618	C_15_H_24_	1518.93	1519	Naphthalene, 1,2,3,5,6,8a-hexahydro-4,7-dimethyl-1-(1-methylethyl)-, (1S-cis)-	54324-03-7	93.9
KMW0604	C_15_H_24_	1498.79	1517	(1S,2E,6E,10R)-3,7,11,11-Tetramethylbicyclo[8.1.0]undeca-2,6-diene	1000414-93-6	97.6
KMW0616	C_15_H_24_	1514.77	1543	(1.alpha.,4a.beta.,8a.alpha.)-1,2,3,4,4a,5,6,8a-octahydro-7-methyl-4-methylene-1-(1-methylethyl)-Naphthalene	5937-11-1	89.9
WMW0013	C_15_H_24_	1465.1	1435	Bicyclosesquiphellandrene	6753-98-6	85.7
XMW0314	C_15_H_24_	1336.67	1431	Elemene isomer	481-34-5	96.2
WMW0018	C_15_H_26_O	1647.86	1580	.tau.-Cadinol	10208-80-7	51.7
KMW0589	C_15_H_24_	1459.35	1467	Humulene	5986-55-0	97.9
KMW0673	C_15_H_26_O	1661.78	1676	.alpha.-Cadinol	30021-74-0	92.1
KMW0606	C_15_H_24_	1452.62	1523	.alpha.-Muurolene	92692-39-2	88.7
XMW0012	C_15_H_24_	1537.57	1538	Naphthalene, 1,2,4a,5,6,8a-hexahydro-4,7-dimethyl-1-(1-methylethyl)-, [1S-(1.alpha.,4a.beta.,8a.alpha.)]-	25246-27-9	97.7
KMW0597	C_15_H_24_	1448.4	1475	.gamma.-Muurolene	29837-12-5	89.3
XMW0045	C_15_H_24_	1493.81	1461	(1R,4R,4aS,8aR)-4,7-Dimethyl-1-(prop-1-en-2-yl)-1,2,3,4,4a,5,6,8a-octahydronaphthalene	77171-55-2	64.8
KMW0596	C_15_H_24_	1442.85	1496	Alloaromadendrene	3856-25-5	96.8
KMW0614	C_15_H_24_	1533.33	1527	β-Patchoulene	514-51-2	74.9
XMW1035	C_15_H_24_O	1579.07	1577	Patchoulol	5986-55-0	83.2
KMW0522	C_15_H_24_	1379.73	1377	(-)-Spathulenol	36577-33-0	83.4
KMW0626	C_15_H_22_	1522.02	1523	Copaene	489-28-1	96.0
XMW0197	C_15_H_24_	1452.17	1440	Naphthalene, 1,2,3,4-tetrahydro-1,6-dimethyl-4-(1-methylethyl)-, (1S-cis)-	489-29-2	76.7
XMW0469	C_15_H_24_	1373.83	1440	(1S,4S,4aS)-1-Isopropyl-4,7-dimethyl-1,2,3,4,4a,5-hexahydronaphthalene	3466-15-7	75.0
WMW0146	C_15_H_24_	1439.19	1443	(1R,3aS,8aS)-7-Isopropyl-1,4-dimethyl-1,2,3,3a,6,8a-hexahydroazulene	29873-99-2	80.2
XMW1408	C_15_H_24_	1415.39	1398	.alpha.-Maaliene	871660-96-7	73.1
WMW0006	C_15_H_22_O	1776.9	1743	1H-Cyclopropa[a]naphthalene, 1a,2,3,3a,4,5,6,7b-octahydro-1,1,3a,7-tetramethyl-, [1aR-(1a.alpha.,3a.alpha.,7b.alpha.)]-	28305-60-4	68.6
KMW0553	C_15_H_24_	1326.98	1425	(3aR,4R,7R)-1,4,9,9-Tetramethyl-3,4,5,6,7,8-hexahydro-2H-3a,7-methanoazulen-2-one	1000131-71-2	46.8
XMW1149	C_15_H_24_	1563.38	1338	.gamma.-Elemene	473-14-3	81.9
D318	C_15_H_24_O	1605.58	1606	(1R,3aS,8aS)-1,4,4,6-Tetramethyl-1,2,3,3a,4,5,7,8-octahydrocyclopenta[c]pentalene	5208-59-3	53.3
XMW1526	C_15_H_24_O	1360.79	1531	.beta.-Oplopenone	1000285-43-6	62.7
XMW3072	C_15_H_24_	1411.21	1481	cis-Z-.alpha.-Bisabolene epoxide	7212-44-4	70.4
KMW0552	C_15_H_24_	1328.93	1417	2,3,4,4a,5,6-hexahydro-1,4a-dimethyl-7-(1-methylethyl)-Naphthalene	39599-18-3	44.2
QWM0001	C_15_H_26_O	1906.33	-	(-)-.beta.-Bourbonene	21391-99-1	88.5
WMW0075	C_15_H_26_O	1384.33	1564	Nerolidol 2		
D449	C_15_H_22_O	1574.49	1766	1,6,10-Dodecatrien-3-ol, 3,7,11-trimethyl-		
KMW0704	C_15_H_20_	1542.28	1859	6-(p-Tolyl)-2-methyl-2-heptenol, trans-		

**Table 2:** Transcriptome analysis of different tissues in *P. cablin*.

Sample	Raw Reads	Clean Reads	Clean Base (G)	Error Rate (%)	Q20 (%)	Q30 (%)	GC Content (%)
PC-F1	51882348	49453672	7.42	0.03	96.97	91.82	47
PC-F2	51493578	49100156	7.37	0.03	96.98	91.83	47.35
PC-F3	52871080	49014140	7.35	0.03	97.27	92.48	47.05
Pc-L1	47467474	42279396	6.34	0.03	98.11	94.37	48.36
Pc-L2	50273198	48043166	7.21	0.03	98.03	94.42	50.33
Pc-L3	48944022	46794004	7.02	0.03	98.04	94.45	49.8
Pc-R1	49688688	47875364	7.18	0.03	97.98	94.23	50.96
Pc-R2	48560362	46803498	7.02	0.02	98.08	94.58	49.73
Pc-R3	47948430	46011708	6.9	0.02	98.18	94.64	49.88
Pc-S1	48140660	46596716	6.99	0.03	98.09	94.34	48.06
Pc-S2	45719632	43067494	6.46	0.02	98.21	94.66	47.48
Pc-S3	47711166	44362466	6.65	0.02	98.22	94.68	47.29
Pc-W1	50698482	47480138	7.12	0.03	97.26	92.21	48.35
Pc-W2	47823910	44074538	6.61	0.02	98.18	94.56	48.26
Pc-W3	44184540	40851022	6.13	0.03	98.07	94.25	48.21

## Data Availability

The original contributions presented in this study are included in the article. Further inquiries can be directed to the corresponding author.

## Abbreviations

ABBREVIATION	FULL TERM	ABBREVIATION	FULL TERM
AACT	Acetyl-CoA C-acetyltransferase	HDS	4-Hydroxy-3-methylbut-2-enyl diphosphate synthase
ACAT	Acetyl-CoA acetyltransferase	HMGCR	3-Hydroxy-3-methylglutaryl-CoA reductase
BPB	4-Phenyl-3-buten-2-one	HMGCS	3-Hydroxy-3-methylglutaryl-CoA synthase
COMT	Caffeic acid O-methyltransferase	HS-SPME	Headspace Solid-Phase Microextraction
CYP450	Cytochrome P450	IPP	Isopentenyl diphosphate
DAMs	Differentially Abundant Metabolites	KEGG	Kyoto Encyclopedia of Genes and Genomes
DEGs	Differentially Expressed Genes	MCT	2-C-Methyl-D-erythritol 4-phosphate cytidylyltransferase
DMAPP	Dimethylallyl diphosphate	MDS	2-C-Methyl-D-erythritol 2,4-cyclodiphosphate synthase
DXR	1-Deoxy-D-xylulose-5-phosphate reductoisomerase	MEP	Methylerythritol phosphate pathway
DXS	1-Deoxy-D-xylulose-5-phosphate synthase	MSEA	Metabolite Set Enrichment Analysis
FPP	Farnesyl diphosphate	MVA	Mevalonate pathway
FPKM	Fragments Per Kilobase per Million mapped reads	MVD	Mevalonate diphosphate decarboxylase
GC-MS	Gas Chromatography-Mass Spectrometry	MVK	Mevalonate kinase
GERD	Germacrene D synthase	NGS	Next-Generation Sequencing
GGPPS	Geranylgeranyl diphosphate synthase	OPLS-DA	Orthogonal Partial Least Squares-Discriminant Analysis
GPP	Geranyl diphosphate	PAL	Phenylalanine ammonia-lyase
TMAP	2,2′,4′,6′-Tetramethylacetophenone	PCA	Principal Component Analysis
TPS	Terpene synthase	PMK	Phosphomevalonate kinase
UV	Unit Variance Scaling	qRT-PCR	Quantitative Real-Time Polymerase Chain Reaction
VIP	Variable Importance in Projection	RI	Retention Index
SRA	Sequence Read Archive	CAS	Chemical Abstracts Service
